# AAV-mediated gene therapy targeting TRPV4 mechanotransduction for inhibition of pulmonary vascular leakage

**DOI:** 10.1063/1.5122967

**Published:** 2019-12-02

**Authors:** Juan Li, Amy M. Wen, Ratnakar Potla, Ezekiel Benshirim, Ariel Seebarran, Maximilian A. Benz, Olivier Y. F. Henry, Benjamin D. Matthews, Rachelle Prantil-Baun, Sarah E. Gilpin, Oren Levy, Donald E. Ingber

**Affiliations:** 1 Wyss Institute for Biologically Inspired Engineering at Harvard University, Boston, Massachusetts 02115, USA; 2 Vascular Biology Program, Boston Children's Hospital and Harvard Medical School, Boston, Massachusetts 02115, USA; 3 Department of Integrative Biology, Harvard College, Cambridge, Massachusetts 02138, USA; 4 Department of Materials Science and Engineering, University of Toronto, Toronto, Ontario M5S 3E4, Canada; 5 Department of Medicine, Boston Children's Hospital and Harvard Medical School, Boston, Massachusetts 02115, USA; 6 Harvard School of Engineering and Applied Sciences, Cambridge, Massachusetts 02139, USA

## Abstract

Enhanced vascular permeability in the lungs can lead to pulmonary edema, impaired gas exchange, and ultimately respiratory failure. While oxygen delivery, mechanical ventilation, and pressure-reducing medications help alleviate these symptoms, they do not treat the underlying disease. Mechanical activation of transient receptor potential vanilloid 4 (TRPV4) ion channels contributes to the development of pulmonary vascular disease, and overexpression of the high homology (HH) domain of the TRPV4-associated transmembrane protein CD98 has been shown to inhibit this pathway. Here, we describe the development of an adeno-associated virus (AAV) vector encoding the CD98 HH domain in which the AAV serotypes and promoters have been optimized for efficient and specific delivery to pulmonary cells. AAV-mediated gene delivery of the CD98 HH domain inhibited TRPV4 mechanotransduction in a specific manner and protected against pulmonary vascular leakage in a human lung Alveolus-on-a-Chip model. As AAV has been used clinically to deliver other gene therapies, these data raise the possibility of using this type of targeted approach to develop mechanotherapeutics that target the TRPV4 pathway for treatment of pulmonary edema in the future.

## INTRODUCTION

Pulmonary edema is a life-threatening condition characterized by abnormal accumulation of intravascular fluid in alveolar air spaces and interstitial tissues of the lungs due to vascular leakage across the alveolar-capillary barrier.^1–4^ Currently, there are no specific therapies to improve vascular permeability, and clinical management relies on providing supportive measures, including diuretics, vasoactive medications, maintenance of adequate nutrition, hemodynamic monitoring, and mechanical ventilation if necessary.^1^ While mechanical ventilation is usually required for the survival of patients with severely compromised lung function, these artificial breathing motions can be detrimental and further compromise the pulmonary vascular barrier as a result of overinflation of the alveoli, a form of “barotrauma” called ventilator-induced lung injury.^5^ Thus, a major challenge in pulmonary medicine is to identify molecular targets unique to lung cells that, if blocked, could prevent the increase in pulmonary vascular permeability, particularly that induced by mechanical distortion.

Transient receptor potential vanilloid 4 (TRPV4) is a promising target for the treatment of pulmonary edema due to its mechanosensitive nature,^6^ along with its roles in regulating endothelial permeability,^7^ epithelial barrier function,^8^ lung myogenic tone,^9^ and lung vascular remodeling in response to hypoxia.^10–12^ TRPV4 ion channels can be activated within 4 ms after mechanical forces are transmitted across cell surface receptors, and mechanical activation of these channels, such as associated with breathing motions or vascular pressure, has been shown to contribute to pulmonary edema progression.^6,13^ While chemical inhibitors of TRPV4 channel activity are known and have been shown to prevent pulmonary vascular leakage,^13,14^ TRPV4 plays a ubiquitous role and is involved in the regulation of diverse bodily functions, including control of serum osmolarity,^15–22^ nociception,^23–26^ bone formation and remodeling,^27–30^ and bladder tone.^31–34^ Therefore, to reduce adverse effects and dose-limiting toxicities from off-target effects of systemic administration of TRPV4 inhibitors,^35^ we explored the possibility of developing a more selective inhibitor of pulmonary vascular leakage that preferentially targets the mechanical signaling mechanism by which physical forces activate TRPV4. We have previously shown that mechanical forces that activate TRPV4 are transferred to it from integrin β1 via the transmembrane protein CD98.^6^ In addition, overexpression of the high homology (HH) domain of CD98 by transfection exerted a dominant negative effect that specifically inhibited mechanical, but not chemical, activation of TRPV4.^36^ However, developing this mechanotransduction-targeted approach into a therapeutic strategy requires a more clinically relevant delivery method.

Adeno-associated virus (AAV) vectors have been used for delivery of gene therapies in the clinic because they provide many advantages, including favorable safety profiles, tailorable tissue tropism, and long-term gene expression,^37^ and their efficacy has been demonstrated in wide-ranging clinical trials, from hemophilia B^38^ to Parkinson's disease.^39^ Thus, we set out to explore whether AAV gene delivery vectors can be used to deliver a gene encoding the CD98 HH domain to demonstrate the feasibility of targeting this mechanotransduction pathway as a way to inhibit pulmonary vascular leakage. We first investigated how AAV serotype and different promoters affect the efficiency of AAV-mediated gene transfer to human pulmonary alveolar epithelial cells (HpAECs) and human primary lung microvascular endothelial cells (HpMVECs) and optimized the transduction efficiency of AAV for these cells. The delivery of the CD98 HH domain with the optimized vectors inhibited mechanical strain-induced activation of TRPV4-dependent responses, including calcium influx and cell realignment. As a proof-of-concept in a more complex biomimetic model, we demonstrated that selective inhibition of mechanical signaling through TRPV4 also suppressed pulmonary barrier leakage in a human Lung Alveolus Chip (Alveolus Chip) that has been previously shown to model multiple human pulmonary diseases with high fidelity, including pulmonary edema and pulmonary thrombosis.^13,40,41^

## RESULTS

### Optimization of AAV vectors for pulmonary cell transduction

Different serotypes of AAV have been reported to have differential tissue-targeting tropisms.^42–45^ While no AAV vectors have been specifically optimized to target human lung alveolar epithelial cells or microvascular endothelial cells, AAV2.5T and AAV2/2 have been previously reported to have enhanced targeting efficiency to human lung airway cells^46^ and other types of human endothelial cells,^47,48^ respectively. AAV2.5T is an AAV chimera resulting from the combination of AAV2 (aa1-128) and AAV5 (aa129-725) with one point mutation (A581T),^40^ while AAV2/2 has the AAV2 genome packaged in an AAV2 capsid.^49^ Thus, we first screened these two AAV vectors using recombinant EYFP-expressing constructs and compared their transduction efficiency in HpAECs and HpMVECs. These primary donor-derived cells contain mixed populations, with HpAECs being composed of both type I and type II alveolar epithelial cells and HpMVECs containing pulmonary endothelial cells from both blood and lymphatic sources.

**FIG. 1.**
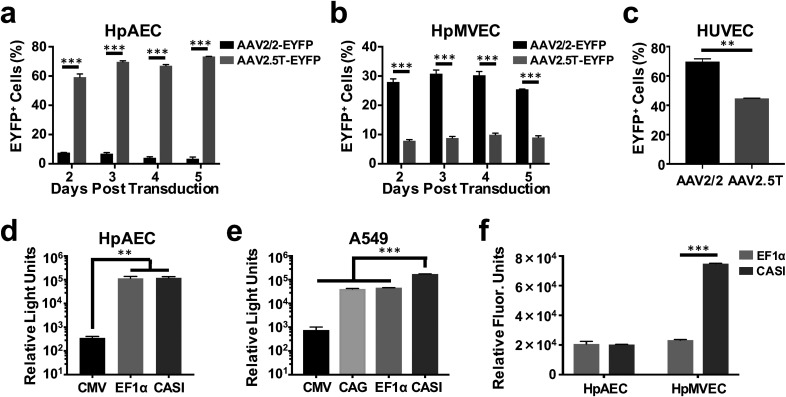
AAV serotypes and promoters affect viral transduction efficiency and transgene expression, respectively. AAV2/2 and AAV2.5T that carry EYFP genes were added to HpAEC (a), HpMVEC (b), or HUVECs (c) at MOI 10 000. Cells were collected 2–5 days after transduction, and the percentage of EYFP-positive cells was analyzed using flow cytometry (n = 2, ^**^ p < 0.01, and ^***^ p < 0.001). AAV2.5T-luciferase with different promoters was added to A549 (d) and HpAEC (e) at MOI 7000. Cells were collected at day two of transduction to measure luciferase activity (n = 3, ^**^ p < 0.01, and ^***^ p < 0.001). (f) AAV2.5T-EYFP with promoters EF1α and CASI was added to HpAEC and HpMVEC at MOI 10 000 and 30 000, respectively. Cells were collected at day three, and the relative fluorescent units of EYFP-positive cells were analyzed by flow cytometry (n = 2, ^***^ p < 0.001).

When added to HpAECs differentiated under air-liquid interface (ALI) culture conditions in Transwells, AAV2.5T (MOI = 10 000) exhibited the highest gene delivery efficiency, with ∼60%–70% being transduced and stable expression sustained for at least 5 days after transduction, whereas only 4%–8% of these alveolar cells were transduced with AAV2/2 [Fig. 1(a)]. Conversely, a higher (25–30%) transduction efficiency was achieved in HpMVECs with AAV2/2 (MOI = 10 000), and this expression was again sustained for at least 5 days, while only 8%–10% of these endothelial cells were transduced with AAV2.5T [Fig. 1(b)]. When these results in primary respiratory cells were benchmarked against the more commonly investigated human umbilical vein endothelial cells (HUVECs), a high gene transfer efficiency of approximately 70% for AAV2/2 (MOI = 10 000) was observed after 3 days, which is consistent with previous literature results.^48^ AAV2.5T was less efficient; however, it still achieved a relatively high transduction efficiency of 45% in HUVECs [Fig. 1(c)].

To further optimize transgene expression, a panel of luciferase-expressing vectors was constructed with several commonly used constitutive promoters (CMV, EF1α, and CASI), and their luciferase activities were compared two days after transduction. While the serotype affects the percentage of cells transduced, once a cell is transduced, it is the promoter that determines the amount of gene expression. From initial testing, it was found that AAV2.5T (MOI = 7000) transduction of HpAECs resulted in stronger transgene expression using either EF1α or CASI promoters, while CMV, the most common promoter used in previous AAV studies, had the lowest gene expression with levels less than 1% of the other promoters [Fig. 1(d)]. Moreover, comparable results were obtained in a second type of human lung alveolar epithelial cells (A549 cells) [Fig. 1(e)]. Again, EF1α and CASI yielded the highest levels of expression, with CASI driving approximately 4-fold higher levels than EF1α. An additional commonly used promoter, CAG, was also investigated with the A549 cells, and although the activity levels were on par with EF1α, it did not result in an improvement over levels observed with EF1α or CASI [Fig. 1(e)].

We therefore proceeded to engineer EYFP-expressing AAV vectors using EF1α and CASI promoters. As we found that AAV2.5T can transduce HpMVECs as well as HpAECS under baseline conditions (albeit at approximately 3-fold lower efficiency) [Fig. 1(b)], we investigated whether integrating EF1α or CASI promoters into this vector could improve this efficiency. When transduced with these new EYFP-expressing AAV2.5T vectors, we found that the two promoters resulted in almost identical transgene expression in HpAECs (MOI = 10 000) when analyzed by flow cytometry 3 days after transduction [Fig. 1(f)]. Interestingly, similar to what we observed in A549 cells, the CASI promoter was 3-fold stronger than the EF1α promoter in HpMVECs (MOI was increased to 30 000 to counterbalance the lower efficiency observed in our initial studies). Due to the combination of the stronger promoter and the higher MOI used, higher EYFP expression in HpMVECs was found, despite lower transduction efficiency of AAV2.5T for these cells relative to HpAECs. Based on these results, the CASI promotor was used for all subsequent studies.

### Disruption of mechanotransduction with AAV-mediated CD98 HH domain delivery

Mechanical activation of TRPV4 ion channels has been implicated in enhanced vascular permeability and pulmonary edema,^13,14^ and overexpression of the CD98 HH domain by transfection with CD98 HH can inhibit the force transfer from β1-integrin to TRPV4 that mediates this response.^36^ As the CD98 HH domain exerts its dominant negative effects on the cytoplasm and transmembrane delivery of large proteins is difficult to pursue clinically, we investigated whether our engineered AAV vectors with high transduction efficiency in human lung cells could be leveraged to deliver the CD98 HH domain and specifically inhibit TRPV4-mediated mechanotransduction.

**FIG. 2.**
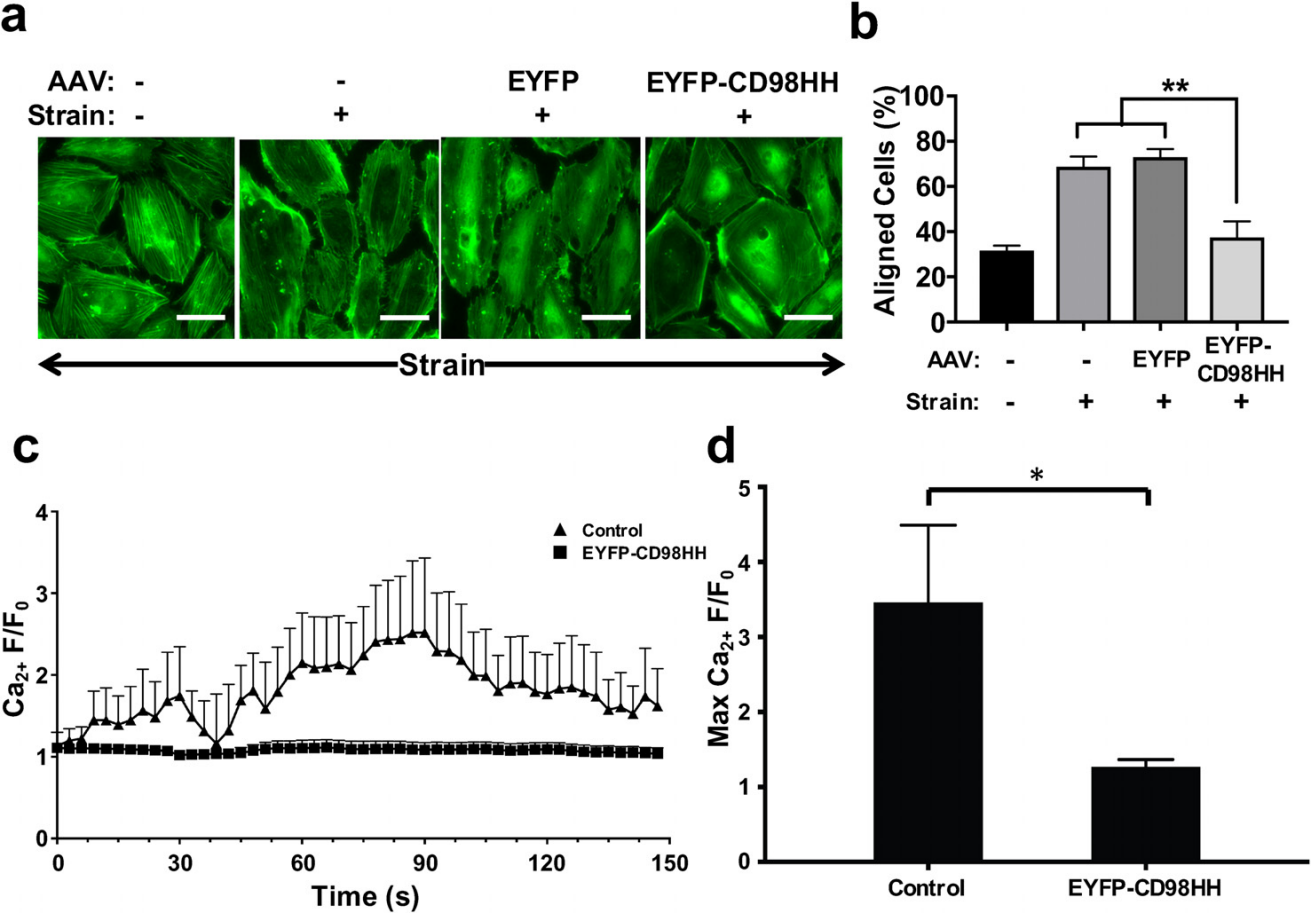
AAV-mediated expression of CD98 HH disrupts cyclic strain-induced cell realignment and calcium response in human pulmonary endothelial cells. (a) Fluorescence images of HpMVECs stained for F-actin after one day of exposure to 15% cyclic strain (1 Hz) in the horizontal direction; cells were transduced with or without AAV2.5T (MOI 10 000) 2 days earlier (bar, 50 *μ*m). (b) Percentage of cells with actin filaments aligned at 90° ± 30° with respect to the direction of cyclic strain (^**^ p < 0.01). (c) Relative changes in cytosolic calcium (Ca_2+_ F/F_0_) in Rhod-3-loaded HUVECs overexpressing high (bottom) or low (top) levels of CD98HH in response to static strain (12% for 15 s, arrow). (d) Average maximum relative changes in cytosolic calcium (Max Ca2+ F/F0) in cells described in **c** (^*^ p < 0.05).

Regulation of endothelial cell reorientation under cyclic mechanical strain is mediated by mechanical activation of TRPV4,^50^ and thus, we used this as an initial readout of mechanotransduction inhibition. Under baseline conditions, approximately 70% of these endothelial cells and their actin cytoskeleton realigned perpendicular to the direction of the applied mechanical strain (15% cyclic strain, 1 Hz) when cultured on flexible extracellular matrix (ECM)-coated culture substrates [Figs. 2(a) and 2(b)]. In contrast, when AAV2.5T vectors encoding the CASI promotor and both the CD98 HH domain and the fluorescent marker EYFP (AAV2.5T-EYFP-CD98HH) were constructed and transduced (MOI = 10 000) in HpMVECs, this strain-induced cell realignment was almost completely prevented, whereas transduction of the control AAV2.5T-EYFP vector had no detectable effect [Figs. 2a and 2(b)].

We then examined the effect of transduction of HpMVECs with AAV2.5T-EYFP-CD98HH on mechanical strain-induced activation of TRPV4-dependent calcium influx, which mediates the cellular reorientation response.^50^ We did not observe an effect of CD98 HH overexpression on the resting level of the Ca2+ concentration in the cytosol. Furthermore, individual cells that expressed higher levels of CD98 HH (quantified by the level of EYFP expression) displayed complete inhibition of mechanically activated calcium signaling, as measured by quantifying Rhod-3 calcium dye influx [Figs. 2(c) and 2(d)]. Overexpression of CD98 HH did not interfere directly with the TRPV4 function, as the addition of the TRPV4 chemical agonist GSK1016790A was able to stimulate calcium influx in these cells (supplementary Fig. S1). Thus, overexpression of the CD98 HH domain through delivery by an AAV vector allows for specific suppression of TRPV4-dependent mechanical signaling pathways in human lung endothelial cells.

### Inhibition of pulmonary vascular leakage in a human Lung Alveolus Chip

An experimental model that has been shown to mimic pulmonary vascular leakage and edema development with high fidelity *in vitro* is the human Lung Alveolus Chip, a 2-channel microfluidic culture device lined with alveolar epithelial cells cultured under ALI, which are interfaced with underlying endothelial cells while experiencing simulated breathing motions and fluid flow mimicking the *in vivo* alveolar microenvironment.^13,51^ While the original Lung Chip utilized A549 lung alveolar epithelial cells and HUVECs, we used an improved model that contains primary HpAECs and HpMVECs^40,41^ in our studies to more directly explore whether AAV vectors encoding the CD98 HH domain could serve as potential mechanotherapeutics for treatment of pulmonary edema in humans.

**FIG. 3.**
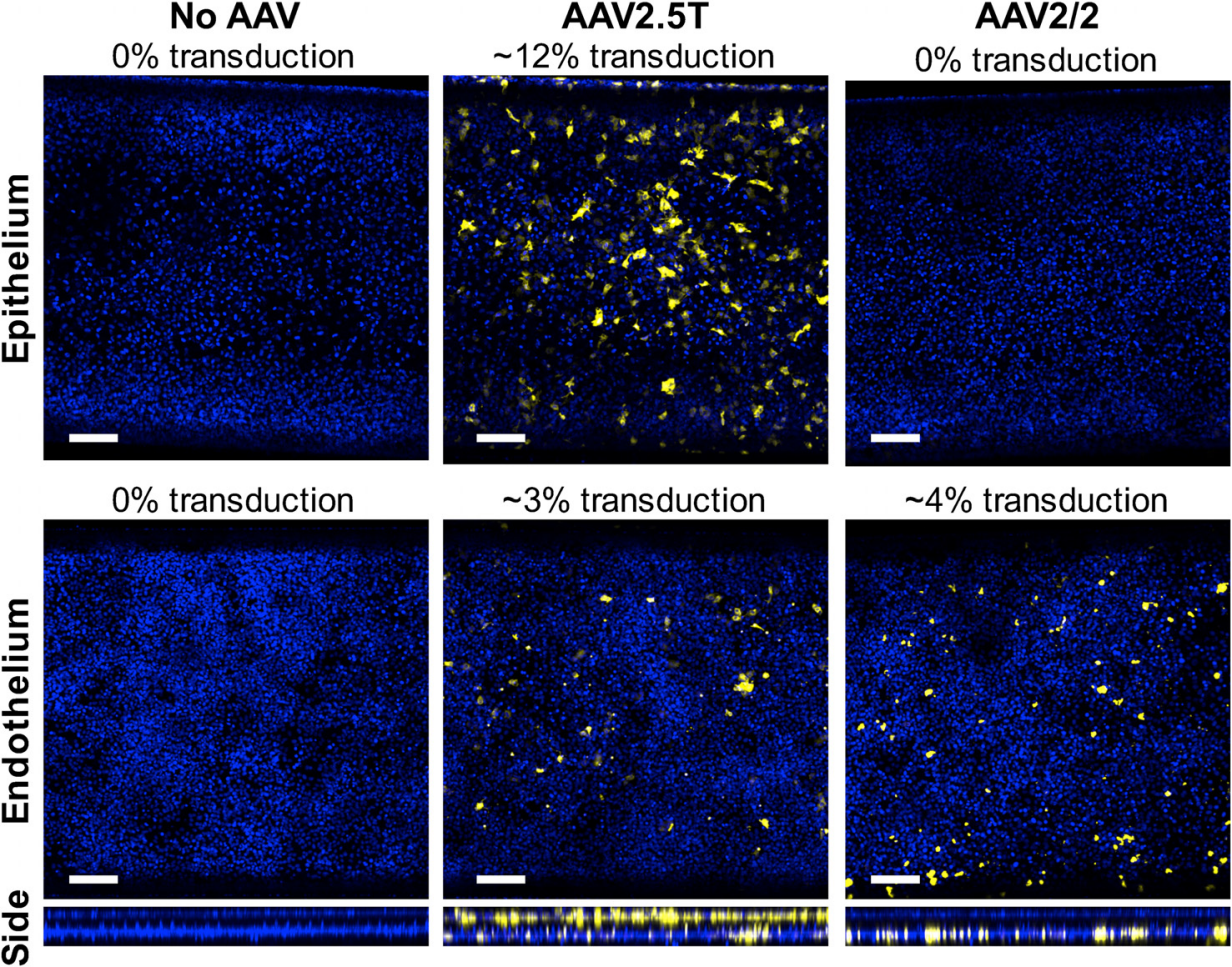
AAV transduction efficiency in Alveolus Chip. AAV-EYFP transduction efficiencies was investigated when AAV2.5T-EYFP (MOI = 10 000) was delivered to HpAEC in the top channel of the chip compared to when AAV2/2-EYFP (MOI= 10 000) was delivered to HpMVEC in the bottom channel of the human Alveolus Chip (left, control; middle, AAV2.5T-EYFP; right, AAV2/2-EYFP). Confocal images were taken at day 6 of AAV transduction.

We first elucidated how the route of delivery influences gene expression in the primary human lung epithelium and endothelium by comparing AAV administration through either the airway or circulatory channels. After culturing the cells on the Alveolus Chip and allowing them to mature following established protocols and timelines^40^ [supplementary Fig. S2(a)], we infused a droplet of medium containing either AAV2.5T-EYFP (MOI = 10 000) to the apical, epithelial channel or AAV2/2-EYFP (MOI = 10 000) to the basal endothelium-lined channel, while exposing the cultures continuously to cyclic strain (10%, 0.2 Hz) to mimic breathing motions. After delivery of the vectors and restoration of the ALI, the entire Alveolus Chip was fed by flowing medium through the endothelium-lined vascular channel to mimic pulmonary capillary perfusion *in vivo*. When we fixed the chips 6 days later and analyzed EYFP expression using confocal microscopy, we observed that gene expression levels were high and sustained for at least 6 days (Fig. 3). Delivery by the apical route was more efficient, with transduction of both HpAECs (∼12%) and HpMVECs (∼3%) compared to delivery by the basal route where only transduction of HpMVECs (∼4%) was achieved. We therefore only administered the vectors apically in subsequent studies.

**FIG. 4.**
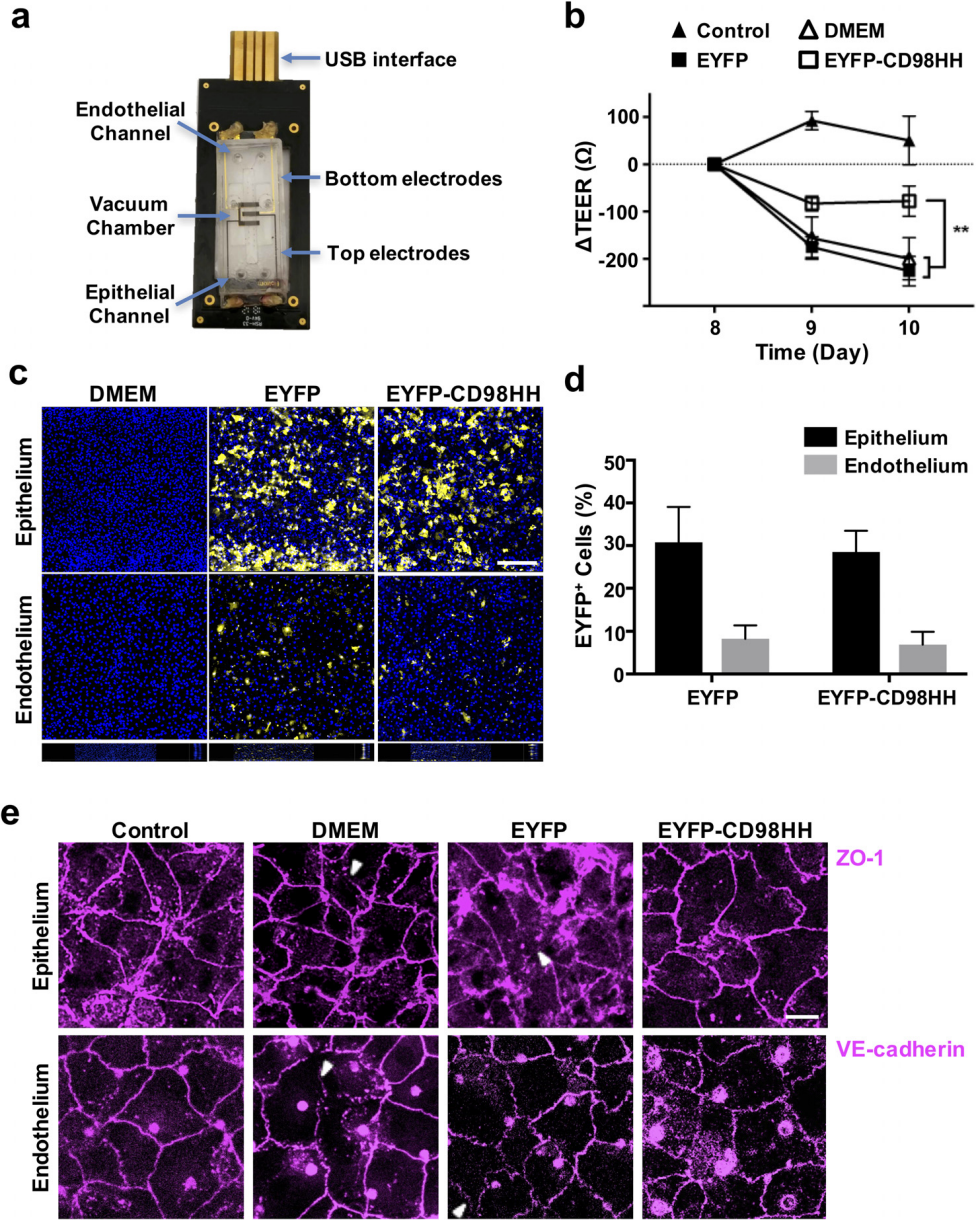
CD98 inhibition prevents pulmonary vascular leakage in a primary human Alveolus Chip. (a) Photograph of the TEER Alveolus Chip setup. (b) Lung barrier permeability reported as changes in TEER in response to mechanical flooding of the apical chamber on day 8 (^**^p < 0.01; Control, n = 5; DMEM, n= 9; AAV2.5T-EYFP, n = 9; AAV2.5T-EYFP-CD98HH, n = 10). (c) Fluorescence micrographs of AAV transduction efficiency of primary epithelial and endothelial cells 3 days after apical delivery for 4 h (MOI = 50 000), displaying EYFP signals from transduced cells (yellow) and DAPI counterstaining (blue) (bar, 200 *μ*m). (d) Percent of cells transduced as quantified by flow cytometry (n = 3). (e) Immunostaining of primary epithelial and endothelial cells for ZO-1 (top) and VE-cadherin (bottom), respectively, to visualize cell-cell junctions 3 days after exposure to mechanical flooding and 10% cyclic strain (bar, 20 *μ*m; arrows indicate the breaks in cell-cell junctions).

Fluid accumulation in the lung alveoli and airways is known to result in the loss of lung surfactant, increased permeability of the alveolar–capillary interface, and decreased lung compliance *in vivo*,^52^ which can influence mechanical signals conveyed across the ECM and integrins that feedback to further promote pulmonary edema. To model this physiologically relevant physical stimulus, we infused the apical channel with medium for 4 h under static conditions on day 8 of Alveolus Chip maturation. For these studies, we used chips that had electrodes embedded into the top and bottom of the channels^53,54^ [Fig. 4(a)], which allowed us to carry out transepithelial electrical resistance (TEER) measurements to quantitatively assess the pulmonary barrier function in real-time. Studies of Alveolus Chip maturation over time confirmed that the TEER chips can sense to changes in barrier integrity. We measured a rise in TEER values on day 5, which is consistent with a significant improvement in the barrier function after the lung epithelium was placed under an ALI, which has been previously shown to promote epithelial differentiation [supplementary Fig. S2(b)]. The TEER levels and hence the barrier were also maintained for at least 2 days after cyclic mechanical strain (10%) was initiated 2 days after shifting the cells to an ALI [supplementary Fig. S2(b)].

After perfusing the epithelial channel with medium, alone, with AAV2.5T-EYFP-CD98HH, or with the control vector AAV2.5T-EYFP (all MOI = 50 000), while maintaining perfusion through the vascular channel, the ALI was restored to the epithelial channel and cyclic strain application was reinitiated. An increase in vascular leakage through the pulmonary barrier was detected by the following day in response to this physical perturbation, as indicated by a decrease in TEER values by ∼200–225 Ohms (Ω) compared to control chips that did not experience fluid immersion [Fig. 4(b); supplementary Fig. S3]. In general, TEER values were ∼800–1000 Ω at maturity, which is in line with what was previously measured for airway epithelial cells that form a pseudostratified epithelium (∼2000 Ω).^53^ Importantly, transduction of the cells with AAV-EYFP-CD98HH encoding the CD98 HH domain significantly inhibited this compromise of the pulmonary barrier function (*p *<* *0.01), while the AAV-EYFP control had no protective response [Fig. 4(b)]. It is important to note that fluorescence microscopic imaging of EYFP expression [Fig. 4(c)] and flow cytometry analysis [Fig. 4(d)] revealed that only ∼30% of HpAECs and ∼10% of HpMVECs were successfully transduced with the AAV vectors under these conditions. Thus, the scale of this protective response is notable given that only a subset of cells expressed the CD98 HH domain.

In addition to the TEER measurements, ZO-1 and VE-cadherin immunofluorescence staining studies were performed for the epithelial and endothelial cells, respectively. For the medium alone and AAV2.5T-EYFP groups, occasional small breaks in cell-cell junctions could be detected, confirming the low-level injury induced by fluid submersion that was measured by TEER [Fig. 4(e)]. Interestingly, although the initial injury was to the epithelium, there was evidence of cross talk between the two cell populations as previously described,^41^ as there were detectable disruptions in the endothelial junctions as well as those in the epithelium [Fig. 4(e)]. Most importantly, fluorescence imaging independently validated that overexpression of CD98 HH protects against this disruption of cell-cell junctions [Fig. 4(e)], which helps to explain its ability to suppress the development of pulmonary vascular leakage as detected by TEER [Fig. 4(b)].

## DISCUSSION

AAV2 is the most commonly used viral vector and was first utilized in Phase I/II clinical trials to target a human lung disease (cystic fibrosis).^55,56^ These studies established safety profiles for AAV2 in humans, but they did not accomplish therapeutic goals due to the low level of gene transfer in lung epithelial cells. Different serotypes of AAV have different tropisms, and it was later discovered that AAV5, rather than AAV2, binds preferentially to the apical surfaces of airway epithelia.^57^ Additionally, AAV5-mediated expression has been reported to persist for at least 12 months in mouse lungs without inducing inflammatory, mechanical, or morphometric changes in the lungs.^58,59^

Here, we demonstrated that AAV2.5T (MOI = 10 000) reached ∼60% transduction of fully differentiated HpAECs. We further optimized AAV2.5T performance by using the CASI promoter to enhance transgene expression, which increased by more than 100-fold compared to that of the original CMV promoter. Based on previous reports that AAV2 is able to transduce HUVECs and brain endothelial cells,^47,48^ we also constructed an endothelium-specific AAV2/2-EYFP vector with a CASI promoter that was able to achieve ∼30%–60% transduction efficiency in two different types of human endothelial cells (HpMVECs and HUVECs).

We then explored whether we could selectively inhibit TRPV4 mechanotransduction by targeting expression of the CD98 HH domain as a therapeutic for pulmonary vascular disease. Using the optimized AAV vectors, we investigated whether the delivery of CD98 HH could be used to rescue the compromised pulmonary barrier function in a human Lung Alveolus Chip, which has been previously shown to model multiple physiological and pathophysiological features of the human lungs, including pulmonary edema.^13,41^ Permeability of the alveolar-capillary interface can be measured *in vitro* by quantifying the passage of fluorescent tracers or directly measuring TEER, as we have previously demonstrated in previous studies using various Organ Chips, including Lung Chips.^13,53,60^ We used TEER measured with embedded electrodes to monitor barrier integrity over time because the TEER method is much more sensitive than the tracer approach (supplementary Fig. S4), and the use of tracer compounds for barrier function measurements can affect transport processes and barrier integrity, especially in Alveolus Chips where the ALI would have to be disrupted for extended periods.^61^

Importantly, by using TEER measurements in the Alveolus Chip, we were able to detect disruption of the pulmonary barrier when the ALI was submerged in liquid medium for 4 h, which is believed to result in a mild mechanical injury due to changes in lung surface tension and compliance.^52^ While AAV delivery of EYFP had no effect, significant protection (>65%) against barrier disruption was conferred when the cells were transduced with CD98 HH. The magnitude of recovery is especially remarkable given that less than 30% of the lung cells were transduced with the AAV vectors based on flow cytometric analysis. The MOI of 50 000 that was used was at the upper limit of the maximum achievable AAV concentration. It would be an interesting avenue for the future to investigate if enhanced therapeutic efficacy could be achieved as AAV serotypes with greater affinity for pulmonary cells are engineered or discovered.

There are chemical inhibitors of TRPV4 activity that are currently in Phase II clinical trials for treatment of pulmonary edema in humans,^62^ which demonstrates the clinical relevance of this target. However, TRPV4 can mediate a plethora of cellular responses, many of which are activated by chemical signals as well as mechanical cues. Thus, it is important to note the significance of the potential to deliver the CD98 HH domain as a highly precise mechanotherapeutic inhibitor that uncouples mechanical activation of TRPV4 from its chemical activation. Using this approach, our results demonstrate that we can minimize the damage to the endothelial-epithelial barrier that occurs from fluid immersion of the airways without affecting TRPV4's chemical signaling function.

Mechanotherapeutic approaches are especially important in the case of patients with acute respiratory distress syndrome (ARDS), who require ventilator support, as fluid accumulation coupled to the stress from the mechanical ventilation can contribute to massive alveolar collapse and perpetuate pulmonary injury.^63^ By targeting the mechanotransduction mechanism that leads to TRPV4 activation in patients on respirators, rather than generically blocking all TRPV4 channel activity, this exacerbation of injury could potentially be avoided without also introducing the possibility of triggering other adverse events from off-target effects of a generalized channel inhibitor. This targeted mechanotherapeutic design strategy also has the potential to protect against additional mechanical injuries that can be induced by other rescue therapies, such as extracorporeal membrane oxygenation (ECMO).^64^ As similar TRPV4-dependent calcium signaling and mechanotransduction have been demonstrated in other cell types (e.g., bovine capillary endothelial cells and human dermal microvascular endothelial cells),^6^ it should be possible to extend this mechanotherapeutic design approach to other cell types and applications. Additionally, one caveat of the present study is that TRPV4 may not be the only channel whose mechanical activation is blocked by CD98 HH; however, this suggests the possibility of identifying other nonchemical inhibitors of such channels, which could lead to additional mechanotherapeutics with greater specificity than direct channel blockers.

## METHODS

### Plasmids and recombinant AAV vector production

Ethics approval was not required. The tested promoters were obtained from the following sources: cytomegalovirus immediate-early promoter (CMV) from pAAV-CMV-luciferase (University of California, Berkeley), chicken β-actin promoter coupled with CMV early enhancer (CAG) from AAV pCAG-FLEX-EGFP-WPRE as a gift from Hongkui Zeng (Addgene Plasmid #51502),^65^ human elongation factor 1α (EF1α) from pAAV-EF1α-EGFP (Wyss Institute at Harvard University), and the CASI promoter, which consists of a CMV enhancer, a chicken β-actin promoter, and a ubiquitin enhancer region,^66^ from pAAV-CASI-Luc2 (Ragon Institute of MGH, MIT, and Harvard). Luciferase and EYFP genes were cloned into the AAV backbone plasmids with different promoters. As described previously,^66,67^ to generate recombinant AAV vectors carrying the transgenes (supplementary Fig. S5), HEK293T cells (ATCC) were cultured in Dulbecco's Modified Eagle Medium (DMEM) supplemented with 10% fetal bovine serum (FBS) and 100 U ml^−1^ penicillin, and the cells were cotransfected with a three-plasmid system: the coding region containing the backbone plasmid, the helper plasmid pHELP (Applied Viromics), and the seed vector pAAV2.5T (University of California, Berkeley) or pAAV2/2 (Massachusetts Eye and Ear) at a ratio of 1:4:8 using the BioT transfection reagent (Bioland Scientific). The culture supernatant was collected, pooled, and filtered through a 0.2 *μ*m filter, and then a 40% polyethylene glycol (PEG) in 2.5 M NaCl solution was added to the supernatant at a volume ratio of 1:4 and gently mixed at 4 °C overnight to precipitate the AAV. The precipitated virus was pelleted at 4000 g for 30 min and resuspended in 10 ml of DMEM. To remove the PEG residue and concentrate the virus, the solution was loaded onto 100 kDa MWCO centrifugal filters (Millipore) and spun at 3220 g at 4 °C until ∼1 ml of retentate remained. Fresh DMEM was added to the filter, and this process was repeated two more times. The final virus solution was about 2 ml total and stored at −80 °C.

### Cell culture

HpAECs and HpMVECs (Cell Biologics) were expanded in tissue culture flasks coated with 0.1% (w/v) gelatin (Sigma) using complete human epithelial/endothelial cell medium (Cell Biologics), respectively, with the FBS concentration reduced to 5% (v/v) for the epithelial cells. HUVECs (Lonza) were cultured in endothelial growth medium supplemented with 5% (v/v) FBS and growth factors (EGM-2; Lonza), while A549 cells (ATCC) were cultured in DMEM/F12 (Gibco) with 10% (v/v) FBS and 1% (v/v) penicillin/streptomycin. The cells were all maintained in an incubator at 37 °C and 5% CO_2_.

### AAV transduction *in vitro*

For plate assays, HpMVECs, HUVECs, and A549 cells were seeded in 6- or 24-well plates, while HpAECs were cultured in 12- or 24-well Transwell plates, treated with 1 *μ*M dexamethasone for 2 days, and introduced to ALI for 4 days before AAV transduction. For chip assays, chips were also allowed to mature at ALI for 4 days (2 days strain) before AAV transduction (see the chip culture section below). AAV vectors were diluted with DMEM and added to cells at MOI ranging from 7000 to 50 000 at 37 °C for 4 h. After removing the virus solution, epithelial cells were washed once with fresh medium before being returned to ALI, while endothelial cells were replaced with fresh medium, and then the cells were cultured for the desired time.

### Flow cytometry

Sample cells were collected by trypsinization and washed with DPBS. After fixing with 2% paraformaldehyde (Electron Microscopy Sciences), cells were stored in 100% methanol at −20 °C for future analysis. On the day of analysis, cells were removed from methanol, washed once with wash buffer (DPBS with 1% normal goat serum and 5 mM NaN_3_), and finally analyzed in wash buffer using a BD LSRFortessa^TM^ flow cytometer.

### Luciferase assay

Two days after AAV transduction, cells were washed with DPBS once and lysed with Glo Lysis Buffer (Promega) at room temperature for five minutes. After transferring to a 96-well plate, the cell lysate was mixed with the Bright-Glo^TM^ Luciferase Assay Reagent (Promega) at a 1:1 volume ratio. Luminescence was then measured using a microplate reader (BioTek).

### Cell realignment and calcium imaging for CD98 HH overexpression experiments

HpMVECs and HUVECs were seeded on collagen IV-coated 6-well UniFlex plates (for cell realignment) or StageFlexer membranes (for calcium imaging) (Flexcell) and transduced 24 h after seeding with AAV2/2-EYFP, AAV2/2-EYFP-CD98HH, or nothing (MOI = 10 000). 1 day post transduction, cells were exposed to uniaxial cyclic strain (15% strain, 1 Hz) for a further 24 h before fixation with 4% paraformaldehyde. F-actin was stained with Alexa Fluor 647 phalloidin (Invitrogen; diluted 1:40) and visualized by confocal microscopy. The percentage of cells aligned 90° ± 30° with respect to the direction of cyclic strain was determined using ImageJ. For calcium imaging, cells were loaded with Rhod-3 calcium dye according to the manufacturer's recommendations, mounted on a StageFlexer device, and imaged using Zeiss Axio Imager 2 during and following 12% static strain for 15 s. 100 cells per membrane were sorted based on EYFP expression into five distinct, equally spaced bins, and analysis was performed comparing intensities from group 5 (high expressers) to group 2 (low expressers). For the group with the TRPV4 chemical agonist GSK1016790A (Tocris), 100 nM GSK1016790A was added 100 s after the start of imaging to induce calcium signaling.

### Confocal imaging

Cells were fixed with 4% paraformaldehyde for 15 min at room temperature and permeabilized with 0.1% Triton X-100 for 5 min. After blocking with 10% goat serum and 0.1% Triton X-100 in DPBS, HpAECs were immunostained with a primary mouse anti-ZO-1 monoclonal antibody (Invitrogen; 1:200) and a secondary goat antimouse IgG1 Alexa Fluor 647 (Invitrogen; 1:1000), while HpMVECs were stained with a primary mouse anti-VE-cadherin antibody (BD Biosciences; 1:200) and a secondary goat antimouse IgG1 Alexa Fluor 594 (Invitrogen; 1:1000) in an incubation buffer consisting of DPBS with 1% goat serum and 0.1% Triton X-100. Nuclei were stained with 4′6-diamidino-2-phenylindole (DAPI) in DPBS for 5 min (0.5 *μ*g ml^−1^). AAV transduction was visualized by EYFP. Images were obtained using a Zeiss TIRF/LSM 710 confocal microscope and analyzed using ImageJ (NIH) and Imaris (Bitplane).

### Chip cell culture

For microfluidic chip cell culture, polydimethylsiloxane (PDMS) chips (Emulate) or TEER chips (see below) were first treated for extracellular matrix (ECM) immobilization using a sulfosuccinimidyl 6–(4′-azido-2′-nitrophenylamino)hexanoate (sulfo-SANPAH) cross-linker (Thermo Fisher Scientific). To do so, channels were sequentially washed with 70% ethanol, water, and 50 mM HEPES, pH 8.0 before incubation with 0.5 mg/ml sulfo-SANPAH in 50 mM HEPES and pH 8.0 with 0.5% DMSO. The sulfo-SANPAH was then photoactivated with a 36 W UV lamp (NailStar) for 30 min. The channels were washed with 50 mM HEPES, pH 8.0 followed by three additional washes with ice-cold Dulbecco's phosphate-buffered saline (DPBS) before incubation with an ECM coating solution (100 *μ*g ml^−1^ human collagen type I (Advanced BioMatrix), 50 *μ*g ml^−1^ human fibronectin (Corning), and 50 *μ*g ml^−1^ laminin nonapeptide [EMD Millipore)] overnight at 4 °C. The following day, the channels were washed once with respective media before seeding of HpAECs (passage 4) and HpMVECs (passage 5) to the apical and basal channels, respectively. Cell seeding was accomplished by first incubating the basal channel with HpMVECs (4 × 10^6^ cells ml^−1^), flipping the devices upside-down, and incubating the chips for 30 min to allow cell attachment. The chips were then returned upright, HpAECs (4 × 10^6^ cells ml^−1^) were introduced to the apical channel, and the devices were cultured in an incubator overnight. The following two days, endothelial growth medium was replaced in the basal channels and 1 *μ*M dexamethasone in epithelial growth medium was introduced in the apical channels to promote tight junction formation and surfactant production.^13,40,68^ ALI was then established by removing the medium from the apical channels, and the basal channels were subjected to continuous perfusion at 60 *μ*l h^−1^ using a peristaltic pump (Ismatec). The perfusion medium for longer-term survival of the cells^40^ was composed of phenol red-free endothelial medium without endothelial cell growth supplement (ECGS) and with reduced FBS (0.4%), reduced EGF (0.01%), and additional CaCl_2_ (1 mM). After 2 days at ALI, physiological breathing motion was initiated by applying vacuum suction to the side chambers of the devices to implement cyclical strain (10% strain, 0.2 Hz).

### TEER chip fabrication

Stretchable polydimethylsiloxane (PDMS) microfluidic chips integrated with electrodes for measuring transepithelial electrical resistance (TEER) were fabricated following protocols similar to those reported in the literature.^53^ In brief, two parallel microchannels served as the air and microvascular channels (800 *μ*m and 200 *μ*m height, respectively, 1 mm width, and 16.7 mm length in the overlapping region), with a 50 *μ*m PDMS membrane in between allowing for epithelial/endothelial cell-cell interactions through 7 *μ*m pores. Electrodes were positioned above and below the channels to allow for a 4-point impedance measurement [see Fig. 4(a)].

### TEER measurements

A PGstat128N potentiostat/galvanostat (Autolab) was used to record four-point impedance spectra in galvanostatic mode over the two-week culture period. The TEER chips were first allowed to equilibrate to room temperature for at least 10 min prior to any measurements. For chips at ALI, 40 *μ*l of room temperature perfusion medium was briefly introduced to the apical channels for the 2 min TEER measurement time before the immediate removal of the medium after each measurement. For each chip, impedance spectra were obtained from 10 Hz to 100 kHz. In the high frequency range (>10 kHz), the impedance curves are dominated by the resistance of the medium, whereas in the low frequency range (<100 Hz), the impedance is dominated by the TEER.^61^ Therefore, reported TEER data were calculated by subtracted medium resistance at 100 kHz from TEER measured at 21.2 Hz.

### Statistical Analysis

All values are expressed as the mean ± standard error of the mean (SEM). All experiments were repeated at least 3 times. Statistical comparisons were performed using Student's t-test for experiments with two conditions or ANOVA for experiments with more than two conditions using GraphPad Prism.

## SUPPLEMENTARY MATERIAL

See the supplementary material for figures of the response of CD98 overexpressing cells to a TRPV4 chemical agonist, Alveolus Chip maturation over time, and the AAV transgene vector map.
